# Uncovering Pluralistic Ignorance to Change Men’s Communal Self-descriptions, Attitudes, and Behavioral Intentions

**DOI:** 10.3389/fpsyg.2018.01344

**Published:** 2018-08-10

**Authors:** Sanne Van Grootel, Colette Van Laar, Loes Meeussen, Toni Schmader, Sabine Sczesny

**Affiliations:** ^1^Center for Social and Cultural Psychology, University of Leuven, Leuven, Belgium; ^2^Fonds Wetenschappelijk Onderzoek, Brussels, Belgium; ^3^Department of Psychology, University of British Columbia, Vancover, BC, Canada; ^4^Institute of Psychology, University of Bern, Bern, Switzerland

**Keywords:** pluralistic ignorance, changing norms, men in HEED, communal attitudes, stereotypes, gender segregation

## Abstract

Gender norms can lead men to shy away from traditionally female roles and occupations in communal HEED domains (Healthcare, Early Education, Domestic sphere) that do not fit within the social construct of masculinity. But to what extent do men underestimate the degree to which other men are accepting of men in these domains? Building on research related to social norms and pluralistic ignorance, the current work investigated whether men exhibit increased communal orientations when presented with the true norms regarding men’s communal traits and behaviors vs. their perceived faulty norms. Study 1 (*N* = 64) revealed that young Belgian men indeed perceive their peers to hold more traditional norms regarding communal and agentic traits than their peers actually hold. Study 2 (*N* = 319) presented young Belgian men with altered norms to manipulate exposure to men’s actual normative beliefs (i.e., what men truly think), their perceived norms (i.e., what men believe other men think), or a no information control. When men were presented with actual rather than perceived norms, they altered their own self-descriptions, future behavioral intentions, and broader gender-related social attitudes in a more communal direction. In particular, men who were presented with information about men’s actual beliefs regarding the compatibility between communal and agentic traits exhibited the strongest movement toward a more communal orientation. The findings show that participants in conditions that uncover pluralistic ignorance adapted their attitudes and behaviors to be more in line with the actual norm: adopting a more communal self-concept, having lower intentions to hide future communal engagement, and supporting more progressive gender-related social change. The results are discussed in terms of influences of norms on men’s communal orientations and broader attitudes toward gender-related social change, and the down-stream implications for increased gender-equality in HEED domains where men remain highly underrepresented.

## Introduction

Gender continues to be a driving force behind men’s and women’s self-selection into some careers and not others. Although real and perceived biases can create obstacles to entry, gender stereotypes can also constrain the interests that men and women have. Moreover, much of the social psychological work on occupational segregation predominantly focuses on women and their underrepresentation in fields often dominated by men, such as science, technology, engineering, and mathematics (i.e., STEM). However, a limited amount of research has focused on the other side of the coin: men’s underrepresentation in fields dominated by women, for example in health care, elementary education, and roles in the domestic sphere (i.e., HEED; Croft et al., 2015). Although the percentage of women in traditionally male-dominated roles has risen somewhat over the last half-century, men’s entry into communal HEED fields traditionally dominated by women has remained fairly low (Croft et al., 2015; Levanon and Grusky, 2016). In HEED fields, in particular, communal qualities are required that embrace the typical female stereotype, focusing on emotional sensitivity and concern for others, such as being kind and considerate, and being understanding and perceptive. On the other hand, in STEM fields, in particular agentic qualities are required that embrace the typical male stereotype, focusing on autonomy and achievement, such as being independent, competent, and results-oriented (Heilman, 2012). Gender differences in the degree to which boys and girls value communion and agency have been found starting already in childhood (Block et al., 2018).

The lack of men in communal fields and domestic roles is concerning. As we will discuss below, when men do engage in communal roles, men, women, children, as well as society as a whole benefit from their active involvement (e.g., Croft et al., 2014). Despite these personal and relational benefits to being communal, those men that have a strong interest in engaging in communal roles may experience societal pressures that keep them out of these roles. Thus, it is of high importance to examine the barriers that men face engaging in communal roles. The current work focuses on how social norms can influence men’s communal attitudes. More specifically, we aim to understand what norms young men have about communal roles, and how these norms can influence young men’s self-descriptions and attitudes toward their own communal engagement.

As noted, despite their underrepresentation in communal roles and behaviors, there are many benefits to men when they do engage in these roles. When engaging in communal roles, men report increased psychological health, higher marital satisfaction (both partners do, Pleck and Masciadrelli, 2004; Knoester et al., 2007; Duckworth and Buzzanell, 2009; Fischer and Anderson, 2012), and higher happiness and overall life satisfaction (e.g., Fleeson et al., 2002; Sheldon and Cooper, 2008; Le et al., 2013, 2018).

Men’s communal engagement is paired with benefits not only for the men themselves, but also for those in their surroundings. Women in dual earner households often face what is called the second shift whereby they engage in more household chores and childcare than their male partner (Milkie et al., 2009; Hochschild and Machung, 2012; Croft et al., 2014). But women who have male partners who are more domestically involved have more flexibility to pursue career ambitions, decreasing the second shift for women. Increased male engagement in domestic roles can thus lift some of the burdens that women face and in turn provide flexibility for women to pursue their career ambitions, closing the gender career achievement gap.

Not only women, but children too experience benefits when men take on communal roles, especially in the domestic sphere. Children show increased cognitive and social development when their fathers engage more in childcare (Marsiglio et al., 2000). Also, girls benefit from their fathers’ involvement in their upbringing by reporting less traditional occupational aspirations and less traditional self-stereotyping (Croft et al., 2014). On a larger societal scale, increasing men’s representation in communal occupations might also provide young boys with salient role models in HEED (e.g., Cochran and Brassard, 1979). For example, having a male elementary school teacher increases the salience of men in that role and may in turn weaken children’s stereotypes (Carrington et al., 2008; Croft et al., 2015). Similar processes are likely to work in other HEED fields, such as in nursing. The shortage of elementary teachers and nurses in many western nations presents an important opportunity to meet these labor shortages by boosting men’s interest in these fields.

Despite these many benefits, men have only increased their engagement in communal roles and behaviors slightly (Bianchi, 2011). Gender norms and roles play an important role in maintaining this inequality for men, as they provide strong ideas about what men are and should be like. Social role theory posits that the roles people enact are influential in shaping the traits they are believed to possess. When biological and historical forces lead men and women to self-segregate into different roles, this role segregation then shapes the stereotypes believed to define gender differences (e.g., Eagly, 1987; Eagly et al., 2000). In this way, men’s historical roles as leaders, protectors, and defenders leads to a stereotype that men relative to women are more competitive, aggressive, strong, and status-seeking. Traits less associated with the male identity are communal traits, such as being compassionate, warm, understanding, etc. (Burgess and Borgida, 1999; Prentice and Carranza, 2002; Rudman and Fairchild, 2004; Diekman and Goodfriend, 2006).

Although stereotypes can be merely descriptive (i.e., this is what men are like), they often become prescriptive norms that play an important role in maintaining traditional male identity by dictating how men ought to be. When men adhere to such norms, their masculine identity is affirmed (e.g., Vandello et al., 2008) and they are socially validated (i.e., role congruity theory, Eagly and Diekman, 2005). Conversely, when men behave in a way that is not in accordance with these norms – for example by portraying more communal and less agentic traits or behaviors – they may experience economic and social penalties (e.g., Rudman and Fairchild, 2004; Moss-Racusin et al., 2010). In order to avoid such penalties, men may seek to adhere to masculine expectations and roles that society imposes, and continuously (re)assert their male identity by engaging in behaviors that conform to the perceived norm of how men should behave (see the social identity approach; Tajfel and Turner, 1979; Turner et al., 1987). This may lead men to refrain from communal behaviors and roles and engage in behaviors that endorse the masculine norm.

Thus far, we have argued that men might avoid communal roles and careers because communal behaviors are incongruent with gender norms, and men may thus expect others to see communal behaviors as “unmanly.” In response, men may avoid or hide communal behaviors and seek to confirm their masculine identity by behaving in ways they think other men in the group behave. Adhering to masculine norms can be done in many positive ways such as working hard, being a good leader, and engaging in sports. Yet research shows that adhering to these norms is also done through risky behaviors such as excessive use of alcohol and drugs (e.g., Locke and Mahalik, 2005; Mahalik et al., 2007; European Union, 2011; SAMHSA, 2015) and risky financial behaviors (Weaver et al., 2013). However, what if men’s perceptions of other men’s beliefs are wrong and men are thus unnecessarily refraining from communal roles and engaging in possibly risky behaviors? What if these behaviors are the result of pluralistic ignorance? Pluralistic ignorance is the (incorrect) belief that one’s personal attitudes are different from the majorities’ attitudes, and thus one goes along with what they think others think (Miller and McFarland, 1991). Pluralistic ignorance thus occurs when people do (not) engage in certain behaviors because they think others would (not) engage in those behaviors (e.g., Miller and McFarland, 1991; Stangor et al., 2001; Sechrist and Stangor, 2005). For example, people’s saving decisions may be influenced by what they think others do or do not save (and may even overshadow their own preference) regardless of whether this is the best financial decision or not. Specifically, people may not think it is important to invest in a 401K pension account plan but when hearing that others are doing so may increase their engagement in those behaviors (Sunstein and Thaler, 2003).

The effects of pluralistic ignorance on behavior has been investigated extensively pertaining to alcohol consumption (e.g., Prentice and Miller, 1993; Schroeder and Prentice, 1998; Suls and Green, 2003). Findings indicate that college students often overestimate the social norm related to drinking behavior, and this leads students to engage in excessive drinking with the goal of fitting in, without necessarily having the goal of excessive consumption (Prentice and Miller, 1996). Related to the current topic, research has shown that there may also be pluralistic ignorance in masculinity norms: men tend to overestimate how aggressive their peers are, overinvest in aggression themselves, and overestimate the extent to which their peers would approve of their aggressive behavior (Bosson et al., 2009; Vandello et al., 2009). We extend this past research by hypothesizing: (a) that men might underestimate other men’s acceptance of communion, and (b) that this underestimation inhibits their engagement in traditionally female communal roles and behaviors.

In the current research, we first examined in Study 1 whether men underestimate the degree to which other men around them value communal behaviors, and to what extent this potentially faulty norm (mis)fits the way they see themselves. By altering these faulty norms in Study 2, we examine whether exposure to different norms about what traits are valued by their peers (i.e., other students at their university) influences men’s own communal self-descriptions, intentions to hide future communal engagement, and broader attitudes toward gender-related social change.

## Study 1

The goals of Study 1 were to establish whether there is pluralistic ignorance regarding what personality traits and characteristics are normative for men and whether such faulty norms do or do not reflect the way men see themselves. Firstly, we expected pluralistic ignorance in communal traits as evidenced by a discrepancy between men’s own communal descriptions of the ideal man and how they think others in their cohort would describe the ideal man. We hypothesized that the ratings of men’s own ideal man would be higher in communion than their peers’ perceptions of the ideal man, i.e., ratings by others in their student and age cohort (Hypothesis 1). We did not have a clear hypothesis for agentic traits. On the one hand, there could be pluralistic ignorance in agentic traits such that men’s own ideal man would be lower in agency than their perception of other’s ideal man (in line with research showing that men tend to overestimate the extent to which their peers approve aggressive behavior; Vandello et al., 2009). On the other hand, there might not be pluralistic ignorance regarding agentic traits since masculine norms are most often communicated in terms of agency, and thus may be more accurately known. Secondly, we expected that this (incorrect) perception of what others expect of a man would provide an unattainable norm for men, as evidenced by a discrepancy between how men describe themselves and how men think their peers describe the ideal man. We hypothesized that men describe themselves as more communal and less agentic than how they think others in their cohort describe the ideal man, suggesting the perception of an unattainable norm (Hypothesis 2).

### Methods

#### Participants

Study 1 was completed by 71 Belgian male university students. We excluded 7 participants who self-identified as not exclusively heterosexual (because they might be subject to different norms; see also Vandello et al., 2008) or who were born before 1990 (and thus did not match the student age cohort). The resulting 64 participants (*M*_age_ = 21.28, *SD* = 2.08) were enrolled in different majors, with most enrolled in engineering (32%) and psychology (32%).

#### Procedure

The protocol was approved by the University of Leuven’s University Social and Societal Ethics Committee. Belgian male university students participated for the chance to win a gift card to a local store popular amongst students. Participants were recruited via social media and through flyers, and were invited to participate in an online study that took approximately 5 min. After providing informed consent as was specified in the ethics application, participants completed the questionnaire which included both demographic questions and the key trait-description measures. Finally, participants were debriefed.

#### Measures

Participants were asked to rate themselves and the ideal man (both from their own and their perception of their peers’ perspective) on a list of 12 agentic traits (e.g., dominant, competent) and 14 communal traits (e.g., warm, dependent) (based on Abele, 2003; Cuddy et al., 2004; see **Appendix 1** for the complete measures). The order of the 26 traits was randomized between participants within each of the three sections.

##### Self-Description

Participants first indicated to what extent the 12 agentic and 14 communal traits described themselves on a scale from 1 – not at all to 7 – very much (α_agentic_ = 0.77 and α_communal_ = 0.81).

##### Own Ideal Man

Participants then were asked to indicate to what extent they thought the same agentic and communal traits described the ideal man on a scale from 1 – not at all to 7 – very much (α_agentic_ = 0.79, α_communal_ = 0.81).

##### Other Ideal Man

Lastly, participants were asked to indicate to what extent they thought these communal and agentic traits described what their peers (i.e., others in their student and age cohort) thought was the ideal man on a scale from 1 – not at all to 7 – very much (α_agentic_ = 0.84, α_communal_ = 0.83).

#### Analyses

The data were analyzed with paired sample *t*-tests examining the difference between participants’ perception of the ideal man and how they thought their peers would describe the ideal man in terms of communion and agency (Hypothesis 1). A second *t*-test compared the difference between participants’ self-description and how they thought their peers would describe the ideal man in terms of communion and agency (Hypothesis 2). A *post hoc* power analysis conducted with G^∗^Power (Faul et al., 2007) indicated that this sample size (*N* = 64) is sufficient to capture a moderate effect size of *r* = 0.30 with power of 76.7%. Power for each separate effect can be found in **Appendix 2**. Results fully replicated when controlling for age, ethnicity, and study major.

In order to make adjustments for multiple comparisons, we applied the Bonferroni correction, in which the critical value of significance was lowered from *p* = 0.05 to *p* = 0.0125 (α/m, m being the number of tests conducted, in this case four tests).

### Results

**FIGURE 1:**
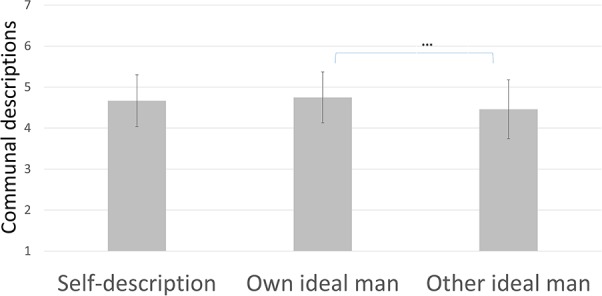
Communal descriptions in Study 1, with SD error bars (^∗∗∗^ indicates *p* < 0.001).

First, we compared participants’ own descriptions of the ideal man with their perceptions of their peers’ descriptions of the ideal man to investigate whether there was indeed pluralistic ignorance. Results (as presented in **Figure 1** and **Table 1**) showed that participants described the ideal man as more communal than they think their peers would describe the ideal man, paired samples *t*_(63)_ = 3.88, *p* < 0.001, *d* = 0.49 (significant at the *p* < 0.0125 level as required by the Bonferroni correction). Thus, the male participants as a group indicated a more communal ideal than they thought their peers would report. Interestingly, men did not describe the ideal man as less agentic than what they believed their peers would report, paired samples *t*_(63)_ = –1.07, *p* = 0.29, *d* = –0.13. This result is consistent with Hypothesis 1, postulating that there is indeed pluralistic ignorance with regard to masculinity norms, and that this pluralistic ignorance is specific to communal traits.

**Table 1:** Means and standard deviations for Study 1 trait descriptions.

	Communal traits	Agentic traits
Self-description	4.67 (0.63)	4.33 (0.69)
Own ideal man	4.75 (0.62)	4.93 (0.63)
Other ideal man	4.46 (0.72)	5.02 (0.73)

**FIGURE 2:**
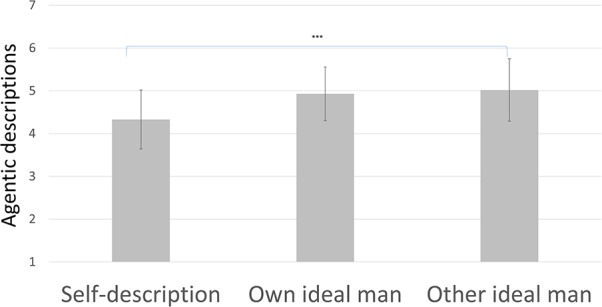
Agentic descriptions in Study 1, with SD error bars (^∗∗∗^ indicates *p* < 0.001).

Second, we compared participants’ self-descriptions with their perception of their peers’ descriptions of the ideal man to investigate whether this perceived norm would be experienced as unattainable. Results (as presented in **Figure 2**) showed a trend such that participants thought that their peers would describe the ideal man as less communal than they on average actually described themselves, paired samples *t*_(63)_ = –1.98, *p* = 0.052, *d* = –0.25, yet this effect did not reach significance. Also, participants thought that their peers would describe the ideal man as more agentic than they on average described themselves, paired samples *t*_(63)_ = –6.32, *p* < 0.001, *d* = –0.79 (significant at the *p* < 0.0125 level as required by the Bonferroni correction). These results suggest that, in line with Hypothesis 2, men perceive that the ideal man is an unattainable norm, especially in terms of agency.

### Discussion

The goal of Study 1 was to establish that men experience pluralistic ignorance and perceive an unattainable norm regarding what traits are deemed desirable and normative for men. Results of this study indicated that indeed there is pluralistic ignorance regarding communal traits as men described the ideal man as more communal than they thought their peers would describe the ideal man. There was no pluralistic ignorance with regard to agentic traits: men’s own perception of the ideal man was not more or less agentic than the perceptions they believed are held by their peers. Conversely, it was mainly agentic traits that provided an unattainable ideal for men (in line with research on precarious manhood and masculinity threat, e.g., Vandello et al., 2008; Bosson and Vandello, 2011), since men described themselves as less agentic than how they believed their peers would describe the ideal man.

Experiencing pluralistic ignorance regarding certain norms reinforces those norms (e.g., Schroeder and Prentice, 1998; Stangor et al., 2001; Sechrist and Milford, 2007). In this case, experiencing pluralistic ignorance regarding what traits are deemed desirable for men is likely to reinforce traditional gender roles and norms of men as needing to be high in agency and low in communion (e.g., Eagly and Steffen, 1984). The findings of Study 1 imply that men may engage in certain behaviors that are not necessarily representative of how they describe the self in order to behave in what they perceive to be a socially desirable or normative manner, even though this may in fact be based on inaccurate information. Adopting traits and behaviors that match a perceived norm but perhaps not the real norm, may thereby actually be reinforcing these (inaccurate) norms, lowering engagement in communal traits and behaviors, and maintaining traditional gender roles and inequalities.

In sum, this study provides the first evidence that men perceive a norm that may not be the actual norm, since men as a group are interested in being more communal than they think their peers expect men to be, and describe the self as less agentic than they think others in their cohort expect men be. Study 2 sets out to examine what happens when we alter these perceived norms.

## Study 2

In Study 2, we set out to examine whether men’s communal attitudes are affected when we alter the perceived norms. Previous research has established the link between normative perceptions and outcomes influenced by pluralistic ignorance (e.g., Stangor et al., 2001; Sechrist and Stangor, 2005). For example, when university students thought the alcohol consumption norm was higher than it actually was, they also tended to drink more. Making explicit this inaccurate perception led participants to moderate their alcohol consumption (Prentice and Miller, 1996). Thus, the goal of Study 2 was to examine the effects of presenting altered norms on men’s attitudes toward communal and agentic self-descriptions, intentions to hide communal engagement, and broader gender-related social change.

Specifically, we constructed five conditions (four experimental conditions and a control condition) in which participants received a norm that was said to be held by their peers. In line with general masculinity norms, the traditional norm condition highlighted that agentic traits are deemed to be most desirable for men to have. The communal norm condition presented the opposite of this, highlighting that communal traits are deemed to be most desirable for men to have. Two further conditions were designed to break the veil of pluralistic ignorance found in Study 1. Specifically, the discrepancy condition highlighted explicitly that while people believe others value especially agency in men, others actually do value communion in men as well. In a fourth compatibility condition, both agentic and communal traits were framed as being important for men to have and compatible with one another. Lastly, in the control condition, no norm was manipulated and thus this functioned as a comparison group reflecting the actual guiding norm as participants perceive it.

The effect of these conditions was investigated on men’s communal and agentic self-descriptions, on their intentions to hide future communal task engagement, and on their broader attitudes toward gender-related social change. This allowed us to examine whether norms reflecting different levels of communion affect how men describe themselves and whether they increase progressive attitudes toward gender-related social change. Hiding future communal task engagement is an important outcome given the evidence that hiding a stigmatized identity can have taxing effects on well-being and social belonging (e.g., Swim and Thomas, 2006; Pachankis, 2007; Newheiser and Barreto, 2014). Also, it is important to investigate under what condition men not only engage more in communal roles but also refrain from hiding such engagement, since hiding maintains the inaccurate norm that men are not communal even when some men actually do engage in communal roles.

We hypothesized that in the two conditions that break the veil of pluralistic ignorance (the discrepancy and compatibility conditions), men will describe themselves in more communal ways without it affecting their agency, report fewer intentions to hide communal behaviors, and hold more progressive attitudes toward gender related social change compared to the control condition. We did not expect differences between the traditional norm condition and the control condition, since the traditional norm condition confirms masculinity norms as present in society. We did not have specific hypotheses about the communal norm condition, but added this condition to compare the effect of merely stressing communal norms to uncovering pluralistic ignorance on men’s self-descriptions, hiding communal engagement, and attitudes toward gender-related social change.

### Methods

#### Participants

In Study 2, participants were 379 Belgian undergraduate men. As in Study 1, 60 participants were excluded as they were born before 1990 or did not self-identify as heterosexual (and are thus potentially subject to different norms, see Vandello et al., 2008), or did not correctly summarize the experimental condition they were in. The resulting 319 participants (*M*_age_ = 21.37, *SD* = 1.95) were enrolled in different majors, with the majority enrolled in engineering (32%) and law (12%).

#### Procedure

The protocol was approved by the University of Leuven’s University Social and Societal Ethics Committee. Participants were invited to complete an online questionnaire on their perceptions of their surroundings and were compensated either with course credit or the chance to win a coupon to a popular store. After agreeing to the informed consent as was specified in the ethics application, participants reported demographics and were randomly assigned to one of the five conditions as described above (please see **Appendix 3** for a more elaborate description of the manipulations): the traditional masculinity norm condition (*n* = 62), the discrepancy condition (*n* = 60), the compatibility condition (*n* = 79), the communal norm condition (*n* = 57), or the control condition (*n* = 61).

In each of the four experimental conditions, participants received an article describing the results of a fictitious study ostensibly conducted at the participants’ university with students of their cohort. Specifically, the study reported students’ beliefs about what traits are valued for an ideal man. Each participant thus received a similar article, but within each article, the traits that were said to be valued differed by condition (as described above). Participants then completed manipulation checks and the dependent variables. Participants in the control condition received no article and instead moved straight to the dependent variables. Lastly, participants moved on to the debriefing, in which they were informed of the research design, including the misleading information, and we explained why this was necessary to test the core hypotheses. Participants were given the contact information of the researcher and of the ethical commission that had approved the research.

#### Measures

A complete overview of all measurement items of this study can be found in **Appendix 4**.

##### Manipulation checks

Participants indicated to what extent the article asserted that communal traits (e.g., vulnerable, dependent, caring, 11 items, α = 0.92) and agentic traits (e.g., ambitious and competent, 7 items, α = 0.90; presented in random order), were valued by their peers on a scale from 1– not at all to 7 – very much (based on Abele, 2003; Cuddy et al., 2004).

##### Communal and agentic self-descriptions

Participants completed scales measuring how they described the self in terms of the same 11 communal (α = 0.82) and 8 agentic traits (α = 0.82; again presented in random order) on a scale ranging from 1 – not at all to 7 – very much (based on Abele, 2003; Cuddy et al., 2004).

##### Hiding of future communal task engagement

This scale assessed to what extent participants thought they would hide their future communal engagement regarding: (a) childcare and (b) household chores from people other than family and friends, specifically: (i) from their future colleagues, (ii) their future boss, and (iii) from strangers (α = 0.90, 6 items), on a scale from 1 – emphasize to 7 – hide. A higher score on this scale is thus indicative of more intent to hide behavior.

##### Attitudes toward gender-related social change

Attitudes toward gender-related social change was measured using an 8 item scale that assessed attitudes regarding changes in society toward gender equality (α = 0.77). Example items include “It is inevitable that men and women will be equal in their work in the future” and “The interests of a typical man will always differ from those of a typical woman, and this will be reflected in the work they choose to do” (reversed). The scale ranged from 1 – strongly disagree 7 – strongly agree, with a higher score on this scale indicating more progressive attitudes regarding social change toward gender equality.

#### Analyses

The data were analyzed using one-way ANOVAs which examined the main effect of condition. Planned pairwise comparisons were conducted with LSD tests. A *post hoc* power analysis conducted with G^∗^Power (Faul et al., 2007) indicated that this sample size was sufficient to capture a moderate effect size of *r* = 0.30 with power of 99.5%. Power for each separate main effect can be found in **Appendix 5**. Results replicated when controlling for age, ethnicity, and study major, with the exception of one effect, as specified below.

### Results

#### Manipulation Checks

Analyses showed that the manipulations were perceived as intended. First, the degree to which participants indicated communal traits had been discussed as valued traits for men in the article differed across the four experimental conditions, *F*_(3,252)_ = 32.01, *p* < 0.001, $\eta_{p}^{2}$ = 0.28. Specifically, planned comparisons showed that those in the traditional norm condition indicated that the article described their peers as valuing communal traits significantly less (*M* = 3.92, *SD* = 1.36) than those in the discrepancy condition (*M* = 5.51, *SD* = 0.89), *p* < 0.001, *d* = –1.29, [–1.96; –1.23]; the compatibility condition (*M* = 4.91, *SD* = 0.96), *p* < 0.001, *d* = –0.87, [–1.33; –0.65]; and the communal norm condition (*M* = 5.49, *SD* = 0.78), *p* < 0.001, *d* = –1.70, [–1.94; –1.20]. Those in the communal norm condition (*M* = 5.49, *SD* = 0.78) and discrepancy condition did not report different levels of communal traits, ns, but reported communal traits as being more valued by those in their cohort than those in the compatibility condition (*M* = 4.91, *SD* = 0.96), *p* < 0.001, *d* = 0.65, [0.26;0.95].

Participants also correctly reported the valued agentic traits for their respective article, *F*_(3, 252)_ = 33.81, *p* < 0.001, $\eta_{p}^{2}$ = 0.29. Planned comparisons showed that those in the traditional norm condition reported agentic traits to be more valued by their peers (*M* = 5.39, *SD* = 0.89) compared to those in the discrepancy condition (*M* = 4.13, *SD* = 1.29), *p* < 0.001, *d* = 1.05, [0.85; 1.68]; the compatibility condition (*M* = 5.00, *SD* = 1.09), *p* = 0.046, *d* = 0.39, [0.01; 0.78]; and the communal norm condition (*M* = 3.48, *SD* = 1.35), *p* < 0.001, *d* = 1.70, [1.49; 2.33]. Those in the communal norm condition indicated agentic traits as being less valued by their peers (*M* = 3.48, *SD* = 1.35) compared to the discrepancy condition, *p* = 0.002, *d* = 0.50, [0.23; 1.07] and the compatibility condition, *p* < 0.001, *d* = 1.27, [1.12; 1.91].

#### Communal and Agentic Self-Descriptions

**FIGURE 3:**
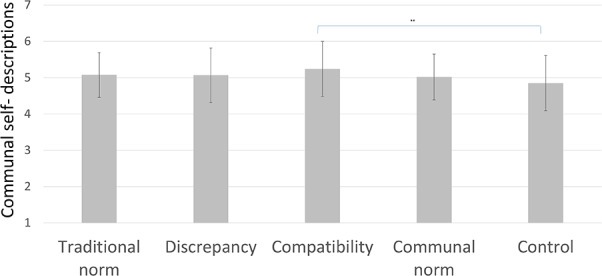
Communal self-descriptions per condition in Study 2, with SD error bars (^∗∗^ indicates *p* < 0.01).

As hypothesized, there was a significant effect of condition on participants’ communal self-descriptions, *F*_(4,314)_ = 2.63, *p* = 0.034, $\eta_{p}^{2}$ = 0.032 (see **Figure 3**). Planned comparisons show that, as expected, men in the compatibility condition described themselves as more communal (*M* = 5.24, *SD* = 0.75) than those in the control condition (*M* = 4.85, *SD* = 0.76), *p* = 0.001, *d* = 0.86, [0.15; 0.63]. There were no significant differences between the other conditions.

There was a marginal effect of condition on agentic self-descriptions, *F*_(4,314)_ = 2.05, *p* = 0.09, $\eta_{p}^{2}$ = 0.025. Planned comparisons indicated that men in the communal norm condition tended to describe themselves as less agentic (*M* = 4.59, *SD* = 0.81) than those in the traditional norm condition (*M* = 4.99, *SD* = 0.81), *p* = 0.01, *d* = –0.50, [–0.70; –0.10]; and marginally less agentic than those in the control condition (*M* = 4.89, *SD* = 0.92), *p* = 0.056, *d* = –0.35, [–0.60; 0.01]. There were no significant differences between the other conditions. However, the effect of condition on agentic self-descriptions disappeared when controlling for study major and the initial effect was only marginal. Therefore, we cannot draw the conclusion that conditions differed in terms of agentic self-descriptions.

#### Hiding Communal Task Engagement

**FIGURE 4:**
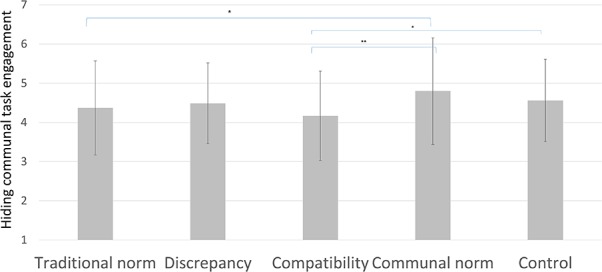
Hiding communal task engagement per condition in Study 2, with SD error bars (^∗^ indicates *p* < 0.05, ^∗∗^ indicates *p* < 0.01).

Next, the extent to which participants expected to hide their future communal engagement from others was investigated. Results show an effect of condition on hiding future communal behaviors from others, *F*_(4,314)_ = 2.71, *p* = 0.030, $\eta_{p}^{2}$ = 0.033 (see **Figure 4**). Planned comparisons revealed that participants in the compatibility condition intended to hide communal engagement less (*M* = 4.17, *SD* = 1.14) than those in the control condition (*M* = 4.56, *SD* = 1.05), *p* = 0.048, *d* = –0.33, [–0.78; 0.00], and also less than those in the communal norm condition (*M* = 4.80, *SD* = 1.36), *p* = 0.002, *d* = –0.51, [–1.03; –0.24]. Unexpectedly, those in the traditional norms condition expected to hide future communal engagement less (*M* = 4.37, *SD* = 1.20) than those in the communal norms condition, *p* = 0.041, *d* = –0.34, [–0.85; –0.02]. There were no significant differences between the discrepancy condition and the other conditions.

#### Attitudes Toward Gender-Related Social Change

**FIGURE 5:**
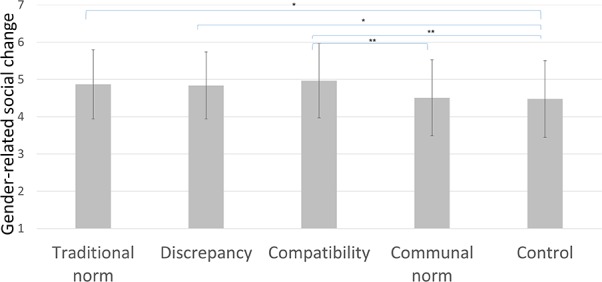
Attitudes toward gender-related social change per condition in Study 2, with SD error bars (^∗^ indicates *p* < 0.05, ^∗∗^ indicates *p* < 0.01).

Finally, there was a main effect of condition on the attitudes toward gender-related social change, *F*_(4,314)_ = 3.35, *p* = 0.010, $\eta_{p}^{2}$ = 0.041 (see **Figure 5**). Specifically, planned comparisons showed that those in the compatibility condition had more progressive attitudes toward gender-related social change (*M* = 4.97, *SD* = 1.00) than those in the control condition (*M* = 4.48, *SD* = 1.03), *p* = 0.004, *d* = 0.49, [–0.82; –0.16], and also than those in the communal norm condition (*M* = 4.51, *SD* = 1.02), *p* = 0.008, *d* = 0.46, [–0.79; –0.12]. Also as expected, those in the discrepancy condition had more progressive attitudes toward gender-related social change (*M* = 4.84, *SD* = 0.90), than those in the control condition (*M* = 4.48, *SD* = 1.03), *p* = 0.045, *d* = –0.35, [0.01; 0.71]. Unexpectedly, those in the traditional norm condition had more progressive attitudes toward gender-related social change (*M* = 4.87, *SD* = 0.93) than those in the control condition (*M* = 4.48, *SD* = 1.03), *p* = 0.027, *d* = –0.37, [0.04; 0.74].

### Discussion

The goal of Study 2 was to examine whether breaking the veil of pluralistic ignorance with regard to norms for men would increase men’s communal self-description, decrease their hiding of future communal task engagement, and make their broader attitudes toward gender-related social change more progressive.

Our findings show that the discrepancy condition (which indicated that while people believe others especially value agency in men, others actually value communion as well) increased participants’ attitudes toward gender-related social change, but it did not affect participants’ self-descriptions or hiding intentions. It could be that the beginning of this manipulation, which highlighted a strong agency prescription for men in society (before uncovering that this was part of pluralistic ignorance amongst their peers) actually made salient a societal masculine norm, decreasing the effectiveness of this condition. The compatibility manipulation (which indicated that both communal and agentic traits were valued in men) had the strongest effects. As expected, in this manipulation participants’ reported more communal self-descriptions without affecting their agentic self-descriptions, less intentions to hide future communal tasks, and more progressive attitudes toward gender-related social change. It thus appears that making salient the actual norm through emphasis on the higher than expected compatibility between agentic and communal traits may be more effective than highlighting the discrepancy between expected and actual norms. This emphasis on the compatibility of communion and agency may allow men to be communal but not at the cost of agency, which is also important for men (e.g., Vandello et al., 2008; Vandello and Bosson, 2012).

Our results also suggest that merely highlighting that men value communal traits may not be sufficient: in the communal condition participants did not report more communal self-descriptions and showed more hiding intentions than in the traditional norm condition. This suggests that when norms stress the value of communion and not agency, men might seek out ways to protect their male identity by hiding communal engagement.

Participants in the traditional norm condition did not differ from those in the control condition regarding their self-descriptions and hiding intentions, suggesting that this traditional norm is similar to their default perception of what the norm is. Unexpectedly, however, those presented with the traditional norm showed more progressive attitudes toward gender-related social change than those in the control condition and less intentions to hide communal self-engagement than those in the communal condition. Perhaps learning of research that confirms the traditional norm provides men with a masculinity affirmation and a sense of certainty as to what the norm is, thus allowing them to report attitudes that are somewhat more progressive (Ridgeway, 2011). This finding may also be caused by the mechanism of paradoxical thinking (Hameiri et al., 2014): when people are presented with opinions they believe but that are phrased more extremely, they tend to show a decrease in their own beliefs. Thus, a presentation of strong masculinity norms may have triggered a counter reaction to such norms in participants.

## General Discussion

Traditional masculine norms are still present even in more progressive societies. Perhaps as a result, men are still highly underrepresented in communal HEED domains such as health care, elementary education, and roles in the domestic sphere (Croft et al., 2015). The very low engagement of men in communal roles and behaviors persists despite increasing insight into the many benefits these careers and roles might have for men’s own well-being (e.g., Fleeson et al., 2002; Sheldon and Cooper, 2008; Le et al., 2013, 2018), but also for their female partner’s upward mobility, children’s aspirations, and for society as a whole (Croft et al., 2014).

To adhere to gender norms, men engage in certain behaviors and roles while avoiding others – in line with what they believe the norm prescribes. Yet, previous research has shown that people may not always have a correct estimate of what the general norm prescribes, which leads them to behave in line with an inaccurate norm; this has been coined “pluralistic ignorance” (Miller and McFarland, 1991; Vandello et al., 2009). The current work aimed to gain more insight into pluralistic ignorance with regard to masculinity norms on communal and agentic traits.

Study 1 established that there is indeed pluralistic ignorance amongst the young men in this sample regarding what traits actually describe the ideal man. Specifically, Study 1 highlighted a difference between these young men’s own perception of the ideal man compared to how they think their peers describe the ideal man. Moreover, this study showed that the perceived norms also prescribe very high agency, higher than the agency men ascribe to themselves. Together, our studies provide a preliminary discovery (see Witte and Zenker, 2017) of pluralistic ignorance in gender norms for men and the potential to increase men’s communal engagement by revealing these erroneous beliefs.

In order to examine the effect of these faulty ideas and the possible correction thereof, Study 2 introduced different norms to test their causal effect on men’s self-description, hiding intentions of communal engagement and attitudes toward gender-related social change. Providing participants with these more accurate depictions of the actual norm indeed had an effect: Highlighting the compatibility between agentic and communal traits seemed especially effective as men exposed to this norm self-described as more communal, showed lower intentions to hide communal engagement, and reported more progressive and broader attitudes toward gender-related social change. This compatibility norm might be powerful because it can allow men to value communion and at the same time maintains the positive value for agentic traits consistent with traditional notions of male identity. In this sense, valuing both agentic and communal traits serves as an affirmation of that identity at the same time that it broadens the identity (Sherman and Cohen, 2002; Derks et al., 2009; Glasford et al., 2009; Spencer-Rodgers et al., 2016). Existing work has also shown that reaffirming important aspects of identity allows exploration of newer aspects of identity traditionally associated with the outgroup (Derks et al., 2006, 2007; Van Laar et al., 2010, 2013). This work thus suggests that valuing agentic in addition to communal aspects may allow men more exploration on the communal side, in that it may decrease possible masculinity threat that is linked to engaging in roles and behaviors that are traditionally female (i.e., precarious manhood, Vandello et al., 2008).

### Limitations and Future Directions

One potential limitation of this work is that participants’ answers in Study 2 may have been affected by demand characteristics – perhaps men could simply have been saying what they just had been told. Although the effects on self-descriptions of traits in Study 2 might be explained in this way (given that the articles mentioned these traits explicitly), it is more difficult to explain the full set of results – including changes in hiding intentions and changes in broader attitudes toward gender-related social change - as demand characteristics. Moreover, demand characteristics are unable to explain the finding that participants in the traditional condition seem to show counter reactions to this norm such that they show lower intentions to hide their communal engagement and they show higher support for gender-related social change. Moreover, the current work is consistent with other studies in which norms were manipulated revealing similar effects (e.g., Schroeder and Prentice, 1998; Stangor et al., 2001; Sechrist and Milford, 2007; Diekman et al., 2013). Further research should investigate whether manipulated norms indeed change the actual perception of norms and lower pluralistic ignorance, and how long these effects persist.

A second possible limitation is the within-participant and cross-sectional nature of Study 1. Such a design was necessary to uncover discrepancies between participants’ own trait descriptions for self or ideal man and these same participants’ perceptions of their peers’ prescriptions for ideal men. Yet, our methods could have given participants insight into the goals of the study. In this case, however, consecutive scales of the same traits would more likely lead to more similar answers on these scales. This would provide a conservative test of Study 1, since it would lead to an underestimation of the expected discrepancies. Also, this concern does not extend to Study 2, which used an experimental manipulation to show that men are affected by varying these norms.

An additional limitation is the relatively small sample size of Study 1. The G^∗^Power analysis for Study 1 indicated that the chance of a Type II error was slightly elevated; β = 0.233 instead of the suggested acceptable probability of β = 0.20 (Cohen, 1992). It is thus important to conduct further studies with large enough samples.

As this is the first work of its kind, these results are a first step and thus can be considered a preliminary discovery (see Witte and Zenker, 2017) of pluralistic ignorance in gender norms for men and the potential to increase men’s communal engagement through uncovering such inaccurate norms. Further research is needed to further investigate the psychological processes at play and to extend these findings. It would be interesting to investigate to what extent the current findings are similar or different in different contexts and samples. The current studies were carried out with male university students pursuing higher education, a sample that is generally associated with more progressive attitudes (e.g., Hoffman and Kloska, 1995). Also, the studies were conducted in Belgium, a cultural context that scores relatively low on gender inequality (UNDP, 2015). Future research could test whether our results generalize to lower educated men and other cultural contexts. While there is no reason to suppose the effects will not generalize, it will be important to replicate these effects in these samples and to consider important moderators. In less progressive samples, it is possible that there is less pluralistic ignorance when men themselves also hold traditional ideals of masculinity (thus showing less of a contrast with perceived ideals held by others). However, it could also be that in these samples, men hold both more traditional ideals of masculinity and perceive stronger ideals held by others so that there is still a relative difference between own and other ideals for men resulting in pluralistic ignorance. Also, different cultures could prescribe different traits that are deemed acceptable or essential for men to hold (for instance, honor is highly valued in some cultures). We would expect that while the content of masculine ideals may differ across cultures, there could still be similar degrees of pluralistic ignorance regarding own and other’s ideals.

Future research could also seek to replicate our findings across age groups. Research shows that as people age, they describe themselves as more communal (Diehl et al., 2004; Roberts et al., 2006). It would be interesting to investigate whether increases in men’s communion as they age are due to the decrease of pluralistic ignorance such that they get a more accurate perception of gender norms over time; or rather that pluralistic ignorance remains, but that with age, people may find it less important to follow gender norms and more important to follow their personal preferences and ideals.

The present research investigated male undergraduate’s peers as an important reference group for normative influence. It would be interesting for future research to also investigate the importance of other groups in setting the norm and influencing men’s communal engagement. For instance, older men, such as the young men’s fathers, or senior men in the workplace may also be important reference groups. Also, women may be an important driving force in setting normative expectations in terms of communal orientations for men, as women benefit from men’s communal investments in the family context (Meeussen et al., 2018).

Also, a field intervention study would be needed to test whether our Study 2 manipulation of creating awareness of pluralistic ignorance may allow men to feel less coerced toward adopting traditional gender roles in real life contexts. There are already some notable projects that aim to increase male engagement in communal roles. For example, through a series of programs and workshops across the world, NGO PROMUNDO (2018) promotes gender equality and encourages gender-related social change, both in educational sessions and campaigns. Based on our findings, it may be interesting to include a component that uncovers pluralistic ignorance in such projects. We would encourage a scientific examination of the effectiveness of these programs and their different components as to inform governmental organizations wishing to promote men taking up paternal leave, increase male representation among elementary school teachers, and increase male representation in nursing.

## Conclusion

The current studies offer the first data consistent with the hypothesis that there exists pluralistic ignorance among men regarding what traits are desirable for an ideal man, and show that uncovering inaccurate beliefs may alter self-descriptions, intentions to hide communal engagement, and broader gender-related social attitudes to better fit with the actual norm. Theoretically, these findings offer initial insights into the underlying normative processes at play in the underrepresentation of men in communal roles. Research such as that presented in this paper can be used to help find more effective ways to address pluralistic ignorance and promote positive gender-related social change.

## Author Contributions

SVG, CVL, LM, TS, and SS contributed to the development of the hypotheses. SVG, CVL, and LM contributed to the data collection. SVG conducted the statistical analyses. All authors contributed to the interpretation of results and the writing of the manuscript.

## Conflict of Interest Statement

The authors declare that the research was conducted in the absence of any commercial or financial relationships that could be construed as a potential conflict of interest.

## References

[B1] Abele A. E. (2003). The dynamics of masculine-agentic and feminine-communal traits: findings from a prospective study. *J. Pers. Soc. Psychol.* 85 768–776. 10.1037/0022-3514.85.4.768 14561129

[B2] Bianchi S. M. (2011). Family change and time allocation in American families. *Ann. Am. Acad. Pol. Soc. Sci.* 638 21–44. 10.1177/0002716211413731

[B3] Block K. Gonzalez A. M. Schmader T. Baron A. S. (2018). Early gender differences in core values predict anticipated family vs. career orientation. *Psychol. Sci.* [Epub ahead of print]. 10.1177/0956797618776942 29932827

[B4] Bosson J. K. Vandello J. A. (2011). Precarious manhood and its links to action and aggression. *Curr. Dir. Psychol. Sci.* 20 82–86. 10.1177/0963721411402669

[B5] Bosson J. K. Vandello J. A. Burnaford R. M. Weaver J. R. Wasti S. A. (2009). Precarious manhood and displays of physical aggression. *Pers. Soc. Psychol. Bull.* 35 623–634. 10.1177/0146167208331161 19202132

[B6] Burgess D. J. Borgida E. (1999). Who women are, who women should be: descriptive and prescriptive gender stereotyping in sex discrimination. *Psychol. Public Policy Law* 5 665–692. 10.1037/1076-8971.5.3.665

[B7] Carrington B. Tymms P. Merrell C. (2008). Role models, school improvement and the “gender gap” - do men bring out the best in boys and women the best in girls? *Br. Educ. Res. J.* 34 315–327. 10.1080/01411920701532202

[B8] Cochran M. M. Brassard J. A. (1979). Child development and personal social networks. *Child Dev.* 50 601–616. 10.2307/1128926

[B9] Cohen J. (1992). A power primer. *Psychol. Bull.* 112 155–159. 10.1037/0033-2909.112.1.155 19565683

[B10] Croft A. Schmader T. Block K. (2015). An underexamined inequality: cultural and psychological barriers to men’s engagement with communal roles. *Pers. Soc. Psychol. Rev.* 19 343–370. 10.1177/1088868314564789 25576312

[B11] Croft A. Schmader T. Block K. Baron A. S. (2014). The second shift reflected in the second generation: do parents’ gender roles at home predict children’s aspirations? *Psychol. Sci.* 25 1418–1428. 10.1177/0956797614533968 24890499

[B12] Cuddy A. J. Fiske S. T. Glick P. (2004). When professionals become mothers, warmth doesn’t cut the ice. *J. Soc. Issues* 60 701–718. 10.1111/j.0022-4537.2004.00381.x

[B13] Derks B. Van Laar C. Ellemers N. (2006). Striving for success in outgroup settings: effects of contextually emphasizing ingroup dimensions on stigmatized group members’ social identity and performance styles. *Pers. Soc. Psychol. Bull.* 32 576–588. 10.1177/0146167205283336 16702152

[B14] Derks B. Van Laar C. Ellemers N. (2007). Social creativity strikes back: improving motivated performance of low status group members by valuing ingroup dimensions. *Eur. J. Soc. Psychol.* 37 470–493. 10.1002/ejsp.375

[B15] Derks B. Van Laar C. Ellemers N. (2009). Working for the self or working for the group: how self – versus group affirmation affects collective behavior in low-status groups. *J. Pers. Soc. Psychol.* 96 183–202. 10.1037/a0013068 19210074

[B16] Diehl M. Owen S. K. Youngblade L. M. (2004). Agency and communion attributes in adults’ spontaneous self-representations. *Int. J. Behav. Dev.* 28 1–15. 10.1080/01650250344000226 18592013 PMC2441921

[B17] Diekman A. B. Goodfriend W. (2006). Rolling with the changes: a role congruity perspective on gender norms. *Psychol. Women Q.* 30 369–383. 10.1111/j.1471-6402.2006.00312.x

[B18] Diekman A. B. Johnston A. M. Loescher A. L. (2013). Something old, something new: evidence of self-accommodation to gendered social change. *Sex roles* 68 550–561. 10.1007/s11199-013-0263-6

[B19] Duckworth J. D. Buzzanell P. M. (2009). Constructing work-life balance and fatherhood: men’s framing of the meanings of both work and family. *Commun. Stud.* 60 558–573. 10.1080/10510970903260392

[B20] Eagly A. H. (1987). Reporting sex differences undergraduate curricula of leading psychology departments. *Am. Psychol.* 42 757–758. 10.1037/0003-066X.42.7.756

[B21] Eagly A. H. Diekman A. B. (2005). “What is the problem? Prejudice as an attitude-in-context,” in *On the Nature of Prejudice: Fifty Years After Allport* eds Dovidio J. F. Glick P. Rudman L. A. (Malden, MA: Blackwell) 19–35.

[B22] Eagly A. H. Steffen V. J. (1984). Gender stereotypes stem from the distribution of women and men into social roles. *J. Pers. Soc. Psychol.* 46 735–754. 10.1037/0022-3514.46.4.735

[B23] Eagly A. H. Wood W. Diekman A. B. (2000). Social role theory of sex differences and similarities: a current appraisal. in *The Development of Social Psychology of Gender* eds Eckes T. Trautner H. M. (Mahwah, NJ: Erlbaum) 123–174.

[B24] European Union (2011). *The State of Men’s Health in Europe.* Available at: https://ec.europa.eu/health/sites/health/files/population_groups/docs/men_health_report_en.pdf.

[B25] Faul F. Erdfelder E. Lang A.-G. Buchner A. (2007). G^∗^Power 3: a flexible statistical power analysis program for the social, behavioral, and biomedical sciences. *Behav. Res. Methods* 39 175–191. 10.3758/BF03193146 17695343

[B26] Fischer J. Anderson V. N. (2012). Gender role attitudes and characteristics of stay-at-home and employed fathers. *Psychol. Men Masc.* 13 16–31. 10.1037/a0024359

[B27] Fleeson W. Malanos A. B. Achille N. M. (2002). An intraindividual process approach to the relationship between extraversion and positive affect: is acting extraverted as “good” as being extraverted? *J. Pers. Soc. Psychol.* 83 1409–1422. 10.1037//0022-3514.83.6.1409 12500821

[B28] Glasford D. E. Dovidio J. F. Pratto E. (2009). I continue to feel so good about us: in-group identification and the use of social identity-enhancing strategies to reduce intragroup dissonance. *Pers. Soc. Psychol. Bull.* 35 415–427. 10.1177/0146167208329216 19141621

[B29] Hameiri B. Porat R. Bar-Tal D. Bieler A. Halperin E. (2014). Paradoxical thinking as a new avenue of intervention to promote peace. *Proc. Natl. Acad. Sci. U.S.A.* 111 10996–11001. 10.1073/pnas.1407055111 25024185 PMC4121792

[B30] Heilman M. E. (2012). Gender stereotypes and workplace bias. *Res. Organ. Behav.* 32 113–135. 10.1016/j.riob.2012.11.003

[B31] Hochschild A. Machung A. (2012). *The Second Shift: Working Parents and the Revolution at Home.* New York, NY: Penguin.

[B32] Hoffman L. W. Kloska D. D. (1995). Parents’ gender-based attitudes toward marital roles and child rearing: development and validation of new measures. *Sex Roles* 32 273–295. 10.1007/BF01544598

[B33] Knoester C. Petts R. J. Eggebeen D. J. (2007). Commitments to fathering and the well-being and social participation of new, disadvantaged fathers. *J. Marriage Fam.* 69 991–1004. 10.1111/j.1741-3737.2007.00426.x

[B34] Le B. M. Impett E. A. Kogan A. Webster G. D. Cheng C. (2013). The personal and interpersonal rewards of communal orientation. *J. Soc. Pers. Relat.* 30 694–710. 10.1177/0265407512466227

[B35] Le B. M. Impett E. A. Lemay E. P. J. Muise A. Tskhay K. O. (2018). Communal motivation and well-being in interpersonal relationships: an integrative review and meta-analysis. *Psychol. Bull.* 144 1–25. 10.1037/bul0000133 29154556

[B36] Levanon A. Grusky D. B. (2016). The persistence of extreme gender segregation in the twenty-first century. *Am. J. Sociol.* 122 573–619. 10.1086/688628

[B37] Locke B. D. Mahalik J. R. (2005). Examining masculinity norms, problem drinking, and athletic involvement as predictors of sexual aggression in college men. *J. Couns. Psychol.* 52 279–283. 10.1037/0022-0167.52.3.279

[B38] Mahalik J. R. Burns S. M. Syzdek M. (2007). Masculinity and perceived normative health behaviors as predictors of men’s health behaviors. *Soc. Sci. Med.* 64 2201–2209. 10.1016/j.socscimed.2007.02.035 17383784

[B39] Marsiglio W. Amato P. Day R. D. Lamb M. (2000). Scholarship on fatherhood in the 1990s and beyond. *J. Marriage Fam.* 62 1173–1191. 10.1111/j.1741-3737.2000.01173.x

[B40] Meeussen L. Van Laar C. Verbruggen M. (2018). Looking for a family man? Norms for men are toppling in heterosexual relationships. *Sex Roles.* 10.1007/s11199-018-0946-0

[B41] Milkie M. A. Raley S. B. Bianchi S. M. (2009). Taking on the second shift: time allocations and time pressures of U.S. parents with preschoolers. *Soc. Forces* 88 487–517. 10.1353/sof.0.0268

[B42] Miller D. T. McFarland C. (1991). “When social comparison goes awry: the case of pluralistic ignorance,” in *Social Comparison: Contemporary theory and research* eds Suls J. Wills T. A. (Hillsdale, NJ: Lawrence Erlbaum) 287–313.

[B43] Moss-Racusin C. A. Phelan J. E. Rudman L. A. (2010). When men break the gender rules: status incongruity and backlash against modest men. *Psychol. Men Masc.* 11 140–151. 10.1037/a0018093

[B44] Newheiser A.-K. Barreto M. (2014). Hidden costs of hiding stigma: ironic interpersonal consequences of concealing a stigmatized identity in social interactions. *J. Exp. Soc. Psychol.* 52 58–70. 10.1016/j.jesp.2014.01.002

[B45] Pachankis J. (2007). The psychological implications of concealing a stigma: a cognitive-affective-behavioral model. *Psychol. Bull.* 133 328–345. 10.1037/0033-2909.133.2.328 17338603

[B46] Pleck J. H. Masciadrelli B. P. (2004). “Paternal involvement by U.S. residential fathers: levels, sources, and consequences,” in *The Role of the Father in Child Development* ed. Lamb M. E. (Hoboken, NJ: John Wiley & Sons Inc) 222–271.

[B47] Prentice D. A. Carranza E. (2002). What women and men should be, shouldn’t be, are allowed to be, and don’t have to be: the contents of prescriptive gender stereotypes. *Psychol. Women Q.* 26 269–281. 10.1111/1471-6402.t01-1-00066

[B48] Prentice D. A. Miller D. T. (1993). Pluralistic ignorance and alcohol use on campus: some consequences of misperceiving the social norm. *J. Pers. Soc. Psychol.* 64 243–256. 10.1037/0022-3514.64.2.243 8433272

[B49] Prentice D. A. Miller D. T. (1996). Pluralistic ignorance and the perpetuation of social norms by unwitting actors. *Adv. Exp. Soc. Psychol.* 28 161–209. 10.1016/S0065-2601(08)60238-5

[B50] PROMUNDO (2018). Available at: https://promundoglobal.org

[B51] Ridgeway C. L. (2011). *Framed by Gender: How Gender Inequality Persists in the Modern World.* Oxford: Oxford University Press 10.1093/acprof:oso/9780199755776.001.0001

[B52] Roberts B. W. Walton K. E. Viechtbauer W. (2006). Patterns of mean-level change in personality traits across the life course: a meta-analysis of longitudinal studies. *Psychol. Bull.* 132 1–25. 10.1037/0033-2909.132.1.1 16435954

[B53] Rudman L. A. Fairchild K. (2004). Reactions to counterstereotypic behavior: the role of backlash in cultural stereotype maintenance. *J. Pers. Soc. Psychol.* 87 157–176. 10.1037/0022-3514.87.2.157 15301625

[B54] SAMHSA (2015). *Results from the 2015 National Survey on Drug Use and Health.* Available at: https://www.samhsa.gov/data/sites/default/files/NSDUH-DetTabs-2015/NSDUH-DetTabs-2015/NSDUH-DetTabs-2015.htm#tab5-6a

[B55] Schroeder C. M. Prentice D. A. (1998). Exposing pluralistic ignorance to reduce alcohol use among college students. *J. Appl. Soc. Psychol.* 28 2150–2180. 10.1111/j.1559-1816.1998.tb01365.x

[B56] Sechrist G. B. Milford L. R. (2007). The influence of social consensus information on intergroup helping behavior. *Basic Appl. Soc. Psychol.* 29 365–374. 10.1080/01973530701665199

[B57] Sechrist G. B. Stangor C. (2005). “Prejudice as social norms,” in *Social Psychology of Prejudce: Historical and Contemporary Issues* eds Crandall C. S. Schaller M. (Lawrence, KS: Lewinian Press) 167–187.

[B58] Sheldon K. M. Cooper M. L. (2008). Goal striving within agentic and communal roles: separate but functionally similar pathways to enhanced well-being. *J. Pers.* 76 415–448. 10.1111/j.1467-6494.2008.00491.x 18399957

[B59] Sherman D. K. Cohen G. L. (2002). Accepting threatning information: self-affirmation and the reduction of defensive biases. *Curr. Dir. Psychol. Sci.* 11 119–123. 10.1111/1467-8721.00182

[B60] Spencer-Rodgers J. Major B. Forster D. E. Peng K. (2016). The power of affirming group values: group affirmation buffers the self-esteem of women exposed to blatant sexism. *Self Identity* 15 413–431. 10.1080/15298868.2016.1145593 27867318 PMC5114007

[B61] Stangor C. Sechrist G. B. Jost J. T. (2001). Changing racial beliefs by providing consensus information. *Pers. Soc. Psychol. Bull.* 27 486–496. 10.1177/0146167201274009

[B62] Suls J. Green P. (2003). Pluralistic ignorance and college student perceptions of gender-specific alcohol norms. *Health Psychol.* 22 479–486. 10.1037/0278-6133.22.5.479 14570531

[B63] Sunstein C. R. Thaler R. H. (2003). *Libertarian Paternalism is not an Oxymoron* Vol. 70 Chicago, IL: The University of Chicago Law Review 1159–1202 10.2307/1600573

[B64] Swim J. Thomas M. A. (2006). “Responding to everyday discrimination: a Synthesis of research on goal directed, self-regulatory coping behaviors,” in *In Stigma and Group Inequality: Social Psychological Approaches* eds Levin S. van Laar C. (Mahwah, NJ: Lawrence Erlbaum Associates Publishers) 126–151.

[B65] Tajfel H. Turner J. C. (1979). “An integrative theory of intergroup conflict,” in *The Social Psychology of Intergroup Relations* eds Austin W. G. Worchel S. (Monterey, CA: Brooks/Cole) 33–47.

[B66] Turner J. C. Hogg M. A. Oakes P. J. Reicher S. D. Wetherell M. S. (1987). *Rediscovering the Social Group: A Self-Categorization Theory.* New York: Basil Blackwell.

[B67] UNDP (2015). *Human Development Report: Work for Human Development.* Available at: http://hdr.undp.org/sites/default/files/2015_human_development_report.pdf

[B68] Van Laar C. Derks B. Ellemers N. (2013). Motivation for education and work in young Muslim women: the importance of value for ingroup domains. *Basic Appl. Soc. Psychol.* 35 64–74. 10.1080/01973533.2012.746609

[B69] Van Laar C. Derks B. Ellemers N. Bleeker D. (2010). Valuing social identity: consequences for motivation and performance in low-status groups. *J. Soc. Issues* 66 602–617. 10.1111/j.1540-4560.2010.01665.x

[B70] Vandello J. A. Bosson J. K. (2012). Hard won and easily lost: a review and synthesis of theory and research on precarious manhood. *Psychol. Men Masc.* 14 101–113. 10.1037/a0029826

[B71] Vandello J. A. Bosson J. K. Cohen D. Burnaford R. M. Weaver J. R. (2008). Precarious manhood. *J. Pers. Soc. Psychol.* 95 1325–1339. 10.1037/a0012453 19025286

[B72] Vandello J. A. Ransom S. Hettinger V. E. Askew K. (2009). Men’s misperceptions about the acceptability and attractiveness of aggression. *J. Exp. Soc. Psychol.* 45 1209–1219. 10.1016/j.jesp.2009.08.006

[B73] Weaver J. R. Vandello J. A. Bosson J. K. (2013). Intrepid, imprudent, or impetuous?: the effects of gender threats on men’s financial decisions. *Psychol. Men Masc.* 14 184–191. 10.1037/a0027087

[B74] Witte E. H. Zenker F. (2017). From discovery to justification: outline of an ideal research program in empirical psychology. *Front. Psychol.* 8:1847. 10.3389/fpsyg.2017.01847 29163256 PMC5663732

