# Supplementary material for: Uncovering Pluralistic Ignorance to Change Men’s Communal Self-descriptions, Attitudes, and Behavioral Intentions
Source: Front Psychol. 2018 Aug 10;9:1344. doi: 10.3389/fpsyg.2018.01344 (PMC6095955; doi:10.3389/fpsyg.2018.01344)
Supplement: Supplementary file 1 [file Presentation_1.pdf]

Appendix 1 - Measures study 1

To what extent do the following traits describe **you**?

		1 – not at all	2	3	4	5	6	7 – very much
Communal traits								
	Dependent							
	Kind							
	Anxious							
	Warm							
	Committed							
	Good-natured							
	Gullible							
	Helpful							
	Trustworthy							
	Vulnerable							
	Emotional							
	Honest							
	Caring							
	Sensitive							
Agentic traits								
	Enterprising							
	Organized							
	Reckless							
	Competent							
	Capable							
	Results-oriented							
	Dominant							
	Tactless							
	Daring to make decisions							
	Efficient							
	Ambitious							
	Punctual							

To what extent do the following traits describe **the ideal man**?

		1 – not at all	2	3	4	5	6	7 – very much
Communal traits								
	Dependent							
	Kind							
	Anxious							
	Warm							
	Committed							
	Good-natured							
	Gullible							
	Helpful							
	Trustworthy							
	Vulnerable							
	Emotional							
	Honest							
	Caring							
	Sensitive							
Agentic traits								
	Enterprising							
	Organized							
	Reckless							
	Competent							
	Capable							
	Results-oriented							
	Dominant							
	Tactless							
	Daring to make decisions							
	Efficient							
	Ambitious							
	Punctual							

To what extent do the following traits describe **the ideal man according to other students at the university of Leuven?**

		1 – not at all	2	3	4	5	6	7 – very much
Communal traits								
	Dependent							
	Kind							
	Anxious							
	Warm							
	Committed							
	Good-natured							
	Gullible							
	Helpful							
	Trustworthy							
	Vulnerable							
	Emotional							
	Honest							
	Caring							
	Sensitive							
Agentic traits								
	Enterprising							
	Organized							
	Reckless							
	Competent							
	Capable							
	Results-oriented							
	Dominant							
	Tactless							
	Daring to make decisions							
	Efficient							
	Ambitious							
	Punctual							

Appendix 2 – G*Power for effects Study 1

Table 1. G*power for each effect found in Study 1

	T statistic	Power
H1: Pluralistic ignorance		
Communal traits	$t(63) = 3.88$, $p < .001$, $d = .49$	99.4%
Agentic traits	$t(63) = -1.07$, *ns*	
H2: Unattainable norm		
Communal traits	$t(63) = -1.98$, $p = .052$, $d = -.25$	54.2%
Agentic traits	$t(63) = -6.32$, $p < .001$, $d = -.79$	100%

Appendix 3 - Manipulations study 2

Traditional norm condition. In this condition, the article discussed the results of the study as showing that the student's cohort valued traditional views of men. Specifically, the students were told that their cohort indicated that men should be very agentic, for example, that men should not only be ambitious and performance oriented, but that they should also show their dominant side.

Communal norm condition. In this condition the article reported the study to have found that the student's cohort valued men to be communal. For example, that men should be understanding and caring, but also warm and vulnerable.

Discrepancy condition. In this condition the article directly made salient the pluralistic ignorance found in Study 1. Participants were told that despite many people thinking that men should show certain types of traits, their cohort actually valued other qualities in men as well. Specifically, this article said that research indicated that students don't think men should only be ambitious, performance oriented and show their dominant side, but also show their caring and emotional side.

Compatibility condition. In this condition participants were shown the article which highlighted the compatibility between the agentic and communal traits. The findings in this article were phrased in such a way, for example, that men's warm, sincere, and sensitive side is valued as much as their athletic, strong, and protective side.

Appendix 4 - Measures study 2

Manipulation checks

How do students at the University of Leuven think a real man should be?

		1 – not at all	2	3	4	5	6	7 – very much
Communal traits								
	Kind							
	Warm							
	Committed							
	Good-natured							
	Helpful							
	Trustworthy							
	Vulnerable							
	Emotional							
	Honest							
	Caring							
	Sensitive							
Agentic traits								
	Enterprising							
	Organized							
	Competent							
	Capable							
	Results-oriented							
	Efficient							
	Ambitious							

Communal and agentic self-description *(self-developed)*
To what extent do the following traits describe you?

		1 – not at all	2	3	4	5	6	7 – very much
Communal traits								
	Kind							
	Warm							
	Committed							
	Good-natured							
	Helpful							
	Trustworthy							
	Vulnerable							
	Emotional							
	Honest							
	Caring							
	Sensitive							
Agentic traits								
	Enterprising							
	Organized							
	Competent							
	Capable							
	Results-oriented							
	Efficient							
	Ambitious							
	Dominant							

Hiding of future communal task engagement *(self-developed)*
To what extent will you try to hide or emphasize <u>the domestic chores</u> you will do from the following people?
These items were then reverse coded, where scoring higher on this scale indicated more hiding intentions.

	1 Hide	2	3	4	5	6	7 Emphasize
Your colleagues							
Your supervisors							
Strangers							

To what extent will you try to hide or emphasize <u>the childcare you will take on at home</u> from the following people?
These items were then reverse coded, where scoring higher on this scale indicated more hiding intentions.

	1 Hide	2	3	4	5	6	7 Emphasize
Your colleagues							
Your supervisors							
Strangers							

Attitudes towards gender-related social change *(self-developed)*

	1 – totally disagree	2	3	4	5	6	7 – totally agree
(R) In groups consisting of men and women, it is more appropriate that men have the leadership positions.							
Traditional roles are disappearing from our society with time.							
It is inevitable that men and women will be equal in their work in the future.							
(R) Men are better leaders.							
(R) Men and women differ in many ways which is why they will never take on the same types of roles.							
(R) A woman’s place is in the household.							
(R) The interests of a typical man will always differ from those of a typical woman and this will be reflected in the work they choose to do.							
(R) Some equality in a marriage is good, but in general the man should be able to make the final decisions in family matters.							

Appendix 5 - G*Power for main effects Study 2

Table 2. G*power for each main effect found in Study 2.

	F statistic	Power
Manipulation checks		
Communal traits	$F(3, 252) = 32.01$, $p < .001$, $\eta_p^2 = .28$	100%
Agentic traits	$F(3,252) = 33.81$, $p < .001$, $\eta_p^2 = .29$	100%
Self-descriptions		
Communal traits	$F(4,314) = 2.63$, $p = .034$, $\eta_p^2 = .032$	73.5%
Agentic traits	$F(4,314) = 2.05$, $p = .09$, $\eta_p^2 = .025$.	60.8%
Hiding communal task engagement		
	$F(4, 314) = 2.71$, $p = .030$, $\eta_p^2 = .033$	75%
Attitudes towards gender-related social change		
	$F(4, 314) = 3.35$, $p = .010$, $\eta_p^2 = .041$.	85%
